# Kyste hydatique pulmonaire: double localisation pulmonaire apicale inhabituelle. A propos d’un cas

**DOI:** 10.11604/pamj.2016.25.159.10357

**Published:** 2016-11-14

**Authors:** Hafsa Sajiai, Mariam Rachidi, Salma Aitbatahar, Hind Serhane, Lamyae Amro

**Affiliations:** 1 Service de Pneumologie, Hôpital ARRAZI, CHU Mohamed VI, Laboratoire PCIM, UCAM, Marrakech, Maroc

**Keywords:** Kyste hydatique, double localisation, apex, Hydatid cyst, double location, apex

## Abstract

Le kyste hydatique (KH) est une pathologie encore endémique dans plusieurs pays notamment dans notre contexte marocain. L’atteinte pulmonaire vient au second plan après l’atteinte hépatique. Elle se caractérise par la richesse des tableaux anotomo-cliniques et la possibilité de localisations multiples dans le parenchyme pulmonaire, prédominant essentiellement aux bases. Nous rapportons le cas de Mr J.M, âgé de 54 ans, admis pour suspicion de kyste hydatique pulmonaire devant une douleur thoracique évoluant depuis 6 mois et un épisode d’hydatidoptysie. La radiographie thoracique a objectivé une image particulière de double localisation apicale illustrant en un même cliché des différents stades évolutifs du kyste hydatique pulmonaire. Le diagnostic était confirmé par la TDM thoracique et la sérologie hydatique. La localisation multiple du kyste hydatique pulmonaire n’est pas une situation rare dans les pays à forte endémie hydatique. Notre cas clinique rapporte une double localisation apicale inhabituelle du kyste hydatique et à des stades évolutifs différents.

## Abstract (en)

Hydatid cyst (KH) is still endemic in several areas of Morocco. Pulmonary involvement is a consequence of liver disease. Hydatid cyst is characterized by diverse anatomical and clinical presentations and by the possibility of multiple locations within the lung parenchyma, predominantly involving pulmonary bases. We report the case of Mr J. M, 54 years old, admitted with suspected pulmonary hydatid cyst based on chest pain lasting for six months and an episode of hydatidoptysis. Chest x-ray objectified a double apical location suggesting different stages of evolution for pulmonary hydatid cyst. The diagnosis was confirmed by thoracic CTscan and hydatid serology. Multiple pulmonary hydatid cyst is not rare in areas in which hydatid disease is highly endemic. Our study reports an unusual double apical location of hydatid cyst at various stages of evolution.

## Introduction

Le Kyste hydatique est une anthropozoonose due au développement de kystes correspondant à la forme larvaire d’un tænia appelé Ecchinococcus granulosis [1]. C’est une anthropozoonose cosmopolite, sévissant en zone d’élevage (ovins, bovins, caprins, porcins, camélidés, équidés, …). Le kyste hydatique pulmonaire (KHP) est la cestodose humaine la plus fréquente. C’est une affection parasitaire qui sévit particulièrement au Moyen-Orient, en Amérique du Sud, en Océanie et dans les pays du pourtour méditerranéen. Sa prévalence au Maroc est très forte. Le poumon représente la deuxième localisation de l’hydatidose après la localisation hépatique. Le diagnostic est habituellement radio clinique, parasitologique (par la mise en évidence de scolex caractéristiques, de crochets, ou de membranes à l’examen direct ou après coupes anatomopathologiques) et immunologique [2]. Le diagnostic immunologique repose sur la recherche d’anticorps spécifiques par des techniques quantitatives (immunofluorescence indirecte, ELISA, hémagglutination) et qualitatives (coélectrosynérèse, immunoélectrophorèse (arc5), immunoempreinte ou western blot). Toutefois, l’interprétation des résultats sérologiques doit rester prudente car un résultat négatif ne permet jamais d’exclure une hydatidose. La localisation pulmonaire droite est généralement prédominante avec une atteinte majeure du lobe inferieur [3]. La localisation apicale a été rarement décrite dans la littérature d’où l’intérêt de notre observation.

## Patient et observation

**Figure 1:**
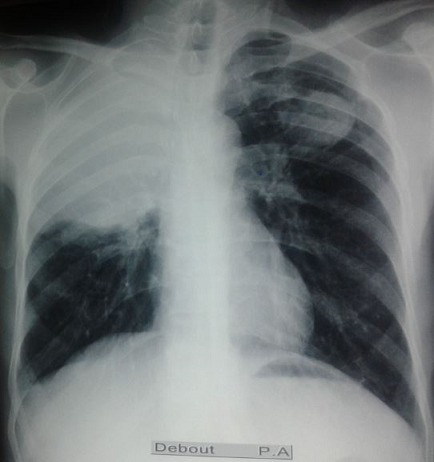
Radiographie thoracique de face montrant une double localisation apicale d’un kyste hydatique

**Figure 2:**
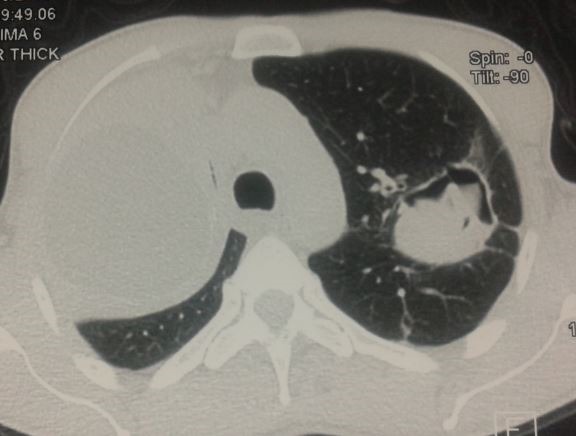
TDM thoracique kyste simple à droite stade I, image en Nénuphar à gauche stade IV (Fenêtre parenchymateuse)

**Figure 3:**
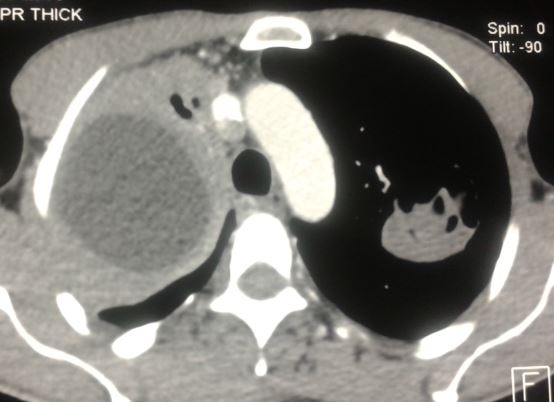
TDM thoracique: kyste simple à droite stade I, image en Nénuphar à gauche stade IV (Fenêtre médiastinale)

**Figure 4:**
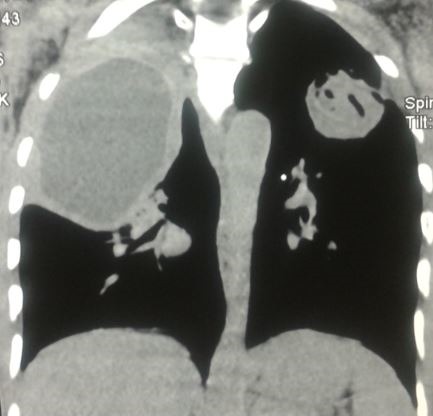
TDM thoracique en reconstitution sagittale montrant les deux localisations du kyste hydatique

Il s’agit de Mr J.M, âgé de 54 ans, agriculteur de profession, non tabagique, résidant en milieu rural, ayant un contact étroit avec les chiens, sans antécédents pathologiques particuliers, qui présente depuis 6 mois une douleur thoracique de l’hémi thorax droit, d’installation progressive, à type de point de côté, à irradiation inter scapulaire postérieure et paroxystique. L’évolution était marquée par l’apparition d’une toux productive tenace ramenant des expectorations verdâtres striées de sang et de 2 épisodes d’hydatidoptysie. Le tout évoluait dans un contexte de conservation de l’état général, de sueurs nocturnes et de sensations fébriles. Un syndrome de condensation apical droit était noté à l’examen clinique. La radiographie du thorax a objectivé une opacité de tonalité hydrique bien limitée occupant les 2/3 supérieurs de l’hémi champs droit et une opacité dense hétérogène sous claviculaire gauche bien limitée surmontée par une clarté formant une image hydro-aérique à niveau irrégulier mamelonné réalisant une image en Nénuphar (Figure 1). Un complément scannographique a été demandé mettant en évidence une volumineuse lésion kystique homogène du lobe supérieur droit mesurant 11.6/ 7.8/ 6.7 cm bien limitée avec atélectasie passive du parenchyme pulmonaire en regard, en rapport avec un KHP non compliqué Stade I. Le poumon gauche était siège d’une masse excavée avec des membranes du segment apico dorsal du culmen réalisant un aspect en nénuphar correspondant à un KHP rompu dans les bronches Stade IV (Figure 2, Figure 3, Figure 4). La bronchoscopie a objectivé une réduction de la lumière de la bronche lobaire supérieure droite, l’etude parasitologique du liquide d’aspiration bronchique n’a pas retrouvé de scolex. La sérologie hydatique a été positive à 12,7 UI. Le reste du bilan biologique a montré une anémie hypochrome microcytaire modérée à 13,8 g/dl, et une légère hyper éosinophilie à 210 El/Ul. Aucune autre localisation associée n’a été retrouvée ni au niveau hépatique ni au niveau cérébrale. Le traitement a consisté en une résection chirurgicale complète en deux temps, d’abord du kyste apical droit sain puis du KHP gauche rompu. Les suites opératoires étaient simples. Le traitement chirurgical était complété par un traitement médical à base d’Albendazole à raison de 10mg/kg démarré avant le geste opératoire et maintenu 3 mois après.

## Discussion

Le kyste hydatique est une pathologie encore endémique au Maroc qui représente un problème de santé publique. La localisation pulmonaire est de loin la plus fréquente des localisations intra-thoraciques. Elle peut être unique ou multiple. L’hydatidose pulmonaire multiple est rencontrée dans 12% des cas [4]. Il s’agit d’une pathologie bénigne mais grave par ses complications mécaniques, infectieuses ou métastatiques. Le KHP peut prendre différents aspects radiologiques selon le stade évolutif. Le KH pulmonaire sain est uni vésiculaire, de volume variable. A la radiographie thoracique, il prend l’aspect d’une opacité de tonalité faible, homogène, de contour net, grossièrement arrondie, en « boulet de canon ». A la TDM thoracique, on retrouve une masse liquidienne, de densité hydrique, bien limitée à paroi lisse et régulière. C’est l’aspect du KH du poumon droit décrit dans notre observation. Au cours de son évolution, le KH peut se compliquer de fissuration, de rupture, de compression, de surinfection voire d’érosions costales. Le KH fissuré prend l’aspect de pneumo kyste avec un croissant gazeux au pôle supérieur du kyste. Rompu, il prend l’aspect d’une image hydro-aérique avec soit un aspect en double arc soit un aspect de membrane flottante. Six stades évolutifs peuvent être décrits en TDM thoracique allant du kyste simple stade I au kyste d’aspect séquellaire stade VI. Un volumineux KH peut comprimer les structures avoisinantes et ainsi, refouler le médiastin ou la coupole diaphragmatique. Il peut également être responsable de trouble de ventilation par compression des bronches. Un aspect de kyste hydatique à paroi épaissie siégeant dans un foyer de condensation parenchymateuse, de bulles d’air en intra kystique ou de niveau hydro-aérique doit orienter vers une surinfection du kyste.

De nombreuses localisations thoraciques inhabituelles de KH ont été décrites dans la littérature. L’atteinte médiastinale reste exceptionnelle faisant l’objet de quelques publications anecdotiques [5]. Dans les pays endémiques, elle présente 0.5% à 2.6% de l’ensemble des localisations thoraciques. Tous les compartiments du médiastin peuvent être affectés. La symptomatologie clinique est variable fonction de la taille, de la topographie et des rapports avec les organes de voisinage. Il est souvent responsable d’un tableau de compression médiastinale du fait du caractère étroit et inextensible du médiastin. En dehors de la compression et de la possibilité d’ouverture dans les bronches, plusieurs signes de souffrance des organes de voisinage ont été rapportés dans la littérature, qu’il s’agit d’érosions vertébrales, de compressions médullaires, d’effractions dans les cavités cardiaques ou dans l’aorte ou de compression de la veine cave supérieure et du sympathique cervical. Le KH pariétal, représente 0.09% à 3.3% des localisations thoraciques. ll peut siéger au niveau costo-vertébrale, sternale ou au niveau des tissus mous. L’hydatidose vertébro-médullaire reste la localisation la plus fréquente et la plus grave de la maladie hydatique dans sa forme osseuse [6]. La greffe parasitaire peut être primitive par voie hématogène, ou secondaire à la rupture spontanée ou peropératoire d’un KH thoracique. Il pose essentiellement problème de diagnostic différentiel avec les lésions tumorales et infectieuses. L’hydatidose thymique est exceptionnelle. L´infestation du thymus semble se faire par voie systémique. L´orientation diagnostique est apportée par l´imagerie thoracique. L’embolie pulmonaire hydatique est très rare, d’évolution imprévisible et pose des problèmes de prise en charge thérapeutique. Elle est due à la rupture de kyste hydatique du cœur droit, plus rarement à la rupture d’un KH hépatique dans la veine cave inférieure ou dans une veine sus hépatique. La localisation primitive artérielle est exceptionnelle. L’évolution est grave pouvant se faire vers l’HTAP et le cœur pulmonaire chronique. Le traitement est idéalement chirurgical mais souvent seul le traitement médical peut être instauré vu la multiplicité des lésions. De rares cas également d’HTAP post embolie pulmonaire ont été décrits. Son pronostic reste réservé malgré le traitement chirurgical [7].

Le KH est unique dans plus de 2/3 des cas, il siège préférentiellement dans le lobe inférieur droit. Récemment, les cas de KH multiples et de localisations multi viscérales sont de plus en plus décrits [8]. Sur le plan physiopathologique, nous distinguons : l’hydatidose primitive multiple en rapport avec des infestations itératives, l’hydatidose secondaire métastatique suite à l’ouverture du KH dans la circulation veineuse, notamment dans la veine cave inférieure et l’hydatidose secondaire bronchogénique qui résulte de la rupture du KH dans les bronches [9]. Concernant la particularité de notre observation, la radiographie thoracique a objectivée une double localisation inhabituellement apicale sans atteinte des lobes basaux confirmée ensuite par la tomodensitométrie. Hors la majorité des KH bilatéraux rapportés dans la littérature siègent préférentiellement dans les lobes basaux et surtout dans la base droite [3, 10]. Le mécanisme de cette double localisation bilatérale du KH serait dans ce cas, des infestations itératives favorisées par la vie en milieu rural.

## Conclusion

L’intérêt de notre étude est essentiellement de mettre en évidence la grande diversité anatomo-clinique du kyste hydatique pulmonaire et de rapporter le cas d’une localisation pulmonaire rare mais non exceptionnelle, apicale bilatérale. Devant la forte prévalence de l’hydatidose dans les pays méditerranéens dont le Maroc, la non spécificité des signes cliniques et sa gravité qui réside essentiellement dans ses complications, le diagnostic du kyste hydatique doit être évoqué et sa prise en charge doit être adaptée. Le volet préventif est capital. Il est basé essentiellement sur la coupure du cycle parasitaire en traitant les chiens et en détruisant les cadavres de moutons infestés.
